# The psychometric properties of the Varieties of Inner Speech Questionnaire-Revised in Hebrew

**DOI:** 10.3389/fpsyg.2022.1092223

**Published:** 2023-01-17

**Authors:** Tal Sabag, Ada H. Zohar, Hamutal Kreiner, Lilac Lev-Ari, Dean Rabinowitz

**Affiliations:** ^1^Ruppin Academic Center, Hadera, Israel; ^2^Lior Zfaaty Center for Suicide and Mental Pain Research, Emek Hefer, Israel; ^3^Faculty of Social Sciences, School of Psychological Sciences, Tel-Aviv University, Tel-Aviv, Israel

**Keywords:** inner speech, executive functions, Hebrew VISQ-R, BRIEF-A, VISQ-R CFA

## Abstract

**Introduction:**

The *Varieties of Inner Speech Questionnaire-Revised* (VISQ-R) is a self-report questionnaire designed to measure characteristics of inner speech. In the current study, we adapted and validated a Hebrew version of VISQ-R. Our first hypothesis was that Confirmatory Factor Analysis (CFA) of the Hebrew VISQ-R would confirm the five subscales replicating the factor structure of the original questionnaire. In addition, building on previous findings that inner speech is involved in tasks that require the executive functions we examined the relationship between VISQ-R and self-reported executive functions questionnaire (BRIEF-A). We hypothesized that correlations between subscales of the Hebrew VISQ-R would reveal covariance between BRIEF-A and some but not all inner speech subscales.

**Methods:**

406 participants completed the Hebrew VISQ-R and 280 of them also completed the BRIEF-A.

**Results:**

As hypothesized, CFA confirmed the factor structure revealing the same 5 subscales reported in the original English version, with acceptable internal reliability. Partial support was found for the hypothesized correlations between VISQ-R and BRIEF-A, with covariance of executive functions with some subscales of inner speech (Evaluative, Other-People and Dialogic), and distinct variance with others (Condensed and Positive).

**Discussion:**

These results indicate that the Hebrew version of the VISQ-R has good psychometric properties and that it can be used in future research. The implications concerning the contribution of inner speech for people with difficulties in executive functions are discussed.

## 1. Introduction

Inner speech has been defined as “the subjective experience of language in the absence of overt and audible articulation.” (Alderson-Day and Fernyhough, 2015, p. 931). The nature of inner speech and its functions are often investigated using experimental manipulations such as eliciting or blocking inner speech and analyzing their effects on different performance aspects. However, the frequency, content, and context of day-to-day inner speech experience are assessed using self-report questionnaires (e.g., Duncan and Cheyne, 1999; Brinthaupt et al., 2009; Alderson-Day et al., 2018). One of the most comprehensive questionnaires developed in recent years for evaluating inner speech is the *Varieties of Inner Speech Questionnaire-Revised* (VISQ-R; McCarthy-Jones and Fernyhough, 2011; Alderson-Day and Fernyhough, 2015; Alderson-Day et al., 2018). As the characteristics of inner speech may vary considerably depending on linguistic and socio-cultural differences (Alderson-Day et al., 2018), it is important to examine its validity in different cultures. Thus, the first aim of the current paper was to develop a Hebrew version of the questionnaire and to examine the psychometric properties of the Hebrew VISQ-R. Furthermore, it has been argued that inner speech is involved in various executive functions (for a review see Alderson-Day and Fernyhough, 2015). Thus, the second goal of the current study was to examine the associations between the Hebrew VISQ-R scores and self-reported executive functions, to provide convergent and divergent validity.

As spontaneous (in contrast to elicited) inner speech cannot be observed or measured directly, many characteristics of its day-to-day experience may be better assessed by the individual’s self-report. Such self-report questionnaires may use an open-format (e.g., Morin et al., 2018), or a list of statements for participants to rate (e.g., Alderson-Day and Fernyhough, 2015). The advantage of open-format questionnaires is that they allow participants to report their inner speech in detail and produce an ecologically valid corpus of inner-speech content. However, as the analysis of such open-format questionnaires is complex, most self-report studies of inner speech use a list of statements and ask participants to rate their agreement with the statements or the frequency of their occurrence. In the current study, we endorse the statement-rating approach, as we focus on the long-term habits of using inner speech, and the functions and nature of inner speech, rather than on its content analysis.

McCarthy-Jones and Fernyhough (2011) composed a 20-item questionnaire called the Varieties of Inner Speech Questionnaire (VISQ). They administered the VISQ to a large sample of college students and conducted exploratory factor analysis. After dropping two items that did not load sufficiently onto the appropriate factor, the VISQ contained 18 items and 4 reliable subscales (derived from a four-factor solution): (1) Dialogic Inner Speech, as in—“When I am talking to myself about things in my mind, it is like I am going back and forward asking myself questions and then answering them;” (2) Condensed Inner Speech as in “My thinking to myself in words is like shorthand notes, rather than full proper grammatical English;” (3) Other-People in Inner Speech as in “I hear the voice of another person in my head. For example, when I have done something foolish, I hear my mother’s voice criticizing me in my mind;” and (4) Evaluative/Motivational Inner Speech as in “I think in inner speech about what I have done and whether it was right or wrong.” The four subscales of the VISQ had structural validity and internal reliability (Alderson-Day and Fernyhough, 2015).

Roebuck and Lupyan (2020) examined the relationship between the VISQ subscales and modes of thought, and found that thinking verbally, as well as mentally picturing written words, correlated positively with the three first subscales of the VISQ. However, thinking in pictures, or mentally manipulating images did not correlate with any of the VISQ subscales, providing convergent and divergent validity for the VISQ. Some external validity for the VISQ subscales is provided by Rosen et al. (2020) and Rosen et al. (2021), who found in a sample of individuals with psychosis and individuals with bi-polar disorder, that the Dialogic, Condensed, and Evaluative inner speech VISQ subscales mediated the relationship between adverse childhood events and auditory hallucinations.

Alderson-Day et al. (2018) further developed the Varieties of Inner Speech Questionnaire. They composed additional items, including items that related to down-regulating negative emotions such as “When I think to myself in words about upsetting things, I can easily change topics in my mind and talk to myself about other things” and items that related to self-encouragement such as “I talk to myself silently in an encouraging way.” The VISQ’s 18 items and these additional items were administered to two large samples online, and an exploratory and a confirmatory factor analysis (CFA) retrieved a 26-item questionnaire, with the 18 original items and 8 new items with five robust subscales, derived from a five-factor solution. The four subscales of the VISQ reemerged as factors with some new items loading onto them, and a new fifth subscale, the Positive/Regulatory subscale composed of four new items completed the factor structure of the revised varieties of inner speech questionnaire (VISQ-R). All five subscales had good internal reliability.

The VISQ-R’s subscales have convergent validity with other self-report scales measuring various aspects of inner speech. For example, Alderson-Day et al. (2018) found that the Evaluative inner speech subscale correlated with self-reported auditory and visual hallucinations, anxiety and depression symptoms, dissociative experiences, and self-esteem. In addition, the Other-People in inner speech subscale correlated with the same self-reported measures, except for self-esteem, while the Dialogic subscale of inner speech correlated only with self-reported auditory and visual hallucination, and with self-esteem. The Condensed and the Positive inner speech subscales did not correlate with any of these self-reported scales. In addition, Rosen et al., (2018) examined the differences between clinical and non-clinical individuals in inner speech and found that individuals with psychosis had greater levels of Other-People, Motivational and Dialogic inner speech, than the non-clinical group. They also found a positive correlation between both Evaluative and Other-People inner speech and auditory verbal hallucinations severity.

More recently, Racy et al. (2022) found positive correlations between the VISQ-R and self-talk frequency, inner experiences such as inner speaking and seeing, use of private speech for self-regulatory and self-reflection purposes, and with subjective perceived frequency of inner speech content and function. In a study that examined the relationship between inner speech and creativity (de Rooij, 2022), the Condensed and Evaluative/Critical subscales of the VISQ-R were found to be negatively correlated with some aspects of creativity, while other subscales did not show any correlations with creative potential. Taken together, these findings show the convergent and divergent validity of the VISQ-R.

The VISQ-R has been adapted to Spanish in a process of translation and independent backtranslation (Perona-Garcelán et al., 2017), and found to have good psychometric properties. The Spanish VISQ-R has five subscales that were found in exploratory factor analysis as well as in CFA. Four are the same as in the English version (Dialogic, Condensed, Other-People, and Evaluative) and the fifth was best named Dialog with Self-positions. All five subscales of the Spanish VISQ-R correlated with self-reported dissociative amnesia, absorption, and depersonalization, as well as with hallucination proneness, in a non-clinical sample.

Inner speech has been linked to executive functions (Alderson-Day and Fernyhough, 2015). Executive functions are a complex cognitive construct used to account for individual differences in the ability to control and regulate thoughts, feelings, and actions, in order to achieve the individual’s goals (Friedman and Miyake, 2017). These functions include cognitive processes such as working memory, attention, and meta-cognition, as well as socio-emotional processes (Perrotta, 2019). Many studies demonstrate the role of inner speech in working memory suggesting that inner speech rehearsal (carried out by the phonological loop) facilitates maintenance of information in memory (e.g., Baddeley, 2003). Other studies suggest that inner speech is involved in task switching, demonstrating that blocking inner-speech increases switch-cost (Miyake et al., 2004). While many of the studies that examined the role of inner-speech used cognitive-behavioral methods such as inner speech rehearsal or elicitation, or blocking inner speech, some studies link between self-reported measures of inner speech and executive functions. For example, it has been found that self-reported increased use of inner speech is linked to reappraisal strategy use in cases of emotional difficulties (Salas et al., 2018). Moreover, there is evidence that inner speech is most frequently used in situations that require self-regulation such as problem-solving, planning, and thinking (Morin et al., 2018), and that using articulatory suppression interferes with self-control (Tullett and Inzlicht, 2010). In addition, Albein-Urios et al. (2021) found that the Evaluative subscale of the VISQ-R moderated the relationship between Autistic Spectrum Disorder (ASD) traits and cognitive reappraisal, suggesting that inner speech has an affective and regulatory role in ASD.

The Behavior Rating Inventory of Executive Function-Adult (BRIEF-A; Roth et al., 2005) is a self-report questionnaire developed to evaluate difficulties in Executive functions. Unlike many of the behavioral tools designed to assess executive functions, the BRIEF-A includes subscales that reflect emotion and regulation which are more likely to be associated with inner speech. For example, previous research found that motivational and evaluative inner speech helped in reducing conflicts effects (Gade and Paelecke, 2019), and that inhibition of inner speech negatively affected the performance in planning tasks (Williams et al., 2012). Hence, in the current study, we will use the Hebrew version of the BRIEF-A (Rotenberg-Shpigelman et al., 2008) to examine the relationship between inner speech and difficulties in executive functions.

The purpose of this study was to assess the validity of the Hebrew version of the VISQ-R. We hypothesized that:

1. Confirmatory Factor Analysis of the Hebrew version of the VISQ-R would reveal five subscales similar to those of the original questionnaire.
2. The correlations between subscales of the Hebrew VISQ-R and self-reported executive functions would reveal covariance between executive functions and some aspects of inner speech but not with all of them. Specifically, we expect that scores on the BRIEF-A will be correlated with VISQ-R subscales related to representation of other people (Dialogic, Other people) and regulation (Evaluative, Positive). By contrast, the Condensed subscale which is more associated with the form of inner speech is not expected to correlate with executive functions.

## 2. Materials and methods

### 2.1. Participants

Participants were recruited either *via* publication on social media for volunteers from the general public (305) or from an undergraduate program of behavioral sciences (101) in an Israeli college. As in the original VISQ-R questionnaire, only participants who completed 80% or more of the items in the VISQ-R were included in the sample. Of the 406 participants that were included, 337 (83.0%) were women, with age ranging between 18 and 66 (M = 30.9; SD = 9.81). 280 (68.96%) participants of the sample also completed the executive functions questionnaire (BRIEF-A).

### 2.2. Materials

#### 2.2.1. Inner speech questionnaire (VISQ-R)

The Varieties of Inner Speech Questionnaire-Revised (VISQ-R; Alderson-Day et al., 2018) was translated into Hebrew (with permission) for this study by a process of translation, independent back-translation, and comparison. The questionnaire includes 26 statements on the frequency of various phenomena of inner speech. Participants were required to rate how frequently they experienced each of these inner speech characteristics on a scale ranging from “Never” (1) to “All the time” (7). Factor analysis on the original English VISQ-R (Alderson-Day et al., 2018) resulted in 5 factors, and in the following confirmatory analysis, these factors were conceptualized as inner speech subscales that reflect different aspects of inner speech: Dialogic, Evaluative (critical), Other-People, Condensed, and Positive (regulatory). The reliability analysis of the original questionnaire based on scores from all 26 items yielded internal reliability of Cronbach’s α > 0.80. The internal reliability of the subscales in the original version was excellent: Dialogic inner speech had an internal reliability of Cronbach’s α = 0.87; Evaluative/Critical inner speech had Cronbach’s α = 0.88; Condensed inner speech had Cronbach’s α = 0.87; Other-People in inner speech had Cronbach’s α = 0.9; and Positive/Regulatory inner speech had Cronbach’s α = 0.80. The Hebrew version of the VISQ-R consisted of the same 26 items as reported in Alderson-Day et al. (2018).

#### 2.2.2. Executive functions questionnaire (BRIEF-A)

Executive functions were assessed by the Hebrew version of the *Behavior Rating Inventory of Executive Function-Adult* (BRIEF-A; Roth et al., 2005). This version includes 75 items describing executive functions as manifested in everyday life and consists of 9 scales: Problems with Inhibition (Cronbach’s α = 0.79), with Task Shifting (Cronbach’s α = 0.79), with Emotional Control (Cronbach’s α = 0.97), with Self-Monitoring (Cronbach’s α = 0.79), with Working Memory (Cronbach’s α = 0.86), with Planning/Organization (Cronbach’s α = 0.87), with Initiating (Cronbach’s α = 0.81), with Task Monitoring (Cronbach’s α = 0.79), and with Organization of Materials (Cronbach’s α = 0.89). In addition, the Global Executive Composite (GEC) reflects overall functioning based on all the items. The participant’s score is the sum of the item scores. The BRIEF-A has excellent ecological validity (Vriezen and Pigott, 2002), and its psychometric properties include internal consistency, structure validity, and discriminant validity (Rotenberg-Shpigelman et al., 2008).

### 2.3. Procedure

The study proposal was approved by the Institution Research Board (IRB; 1.6.2021). The questionnaires were administered online *via* Qualtrics© survey application. Participants confirmed their consent after a brief presentation of the study’s goals and requirements. All participants completed the Hebrew VISQ-R. Following the Hebrew VISQ-R, 305 participants were presented with the BRIEF-A—Executive functions questionnaire, and 280 of these individuals completed at least 80% of the items. The data were collected anonymously and downloaded into SPSS without personal information. The data were examined for completeness and then analyzed in SPSS25 and in AMOS23.

## 3. Results

The results consist of two analyses. First, we present CFA and the reliabilities and inter-correlations of the subscales. These analyses were designed to examine the consistency of constructs structured according to their theoretical or empirical conceptualization, based on the 5 factors reported in Alderson-Day et al. (2018) for the English VISQ-R. Second, we present a correlation analysis of VISQ-R and BRIEF-A executive functions questionnaire aimed to evaluate the convergent and divergent validity of the Hebrew VISQ-R.

### 3.1. Confirmatory factor analysis of the Hebrew VISQ-R

**Figure 1:**
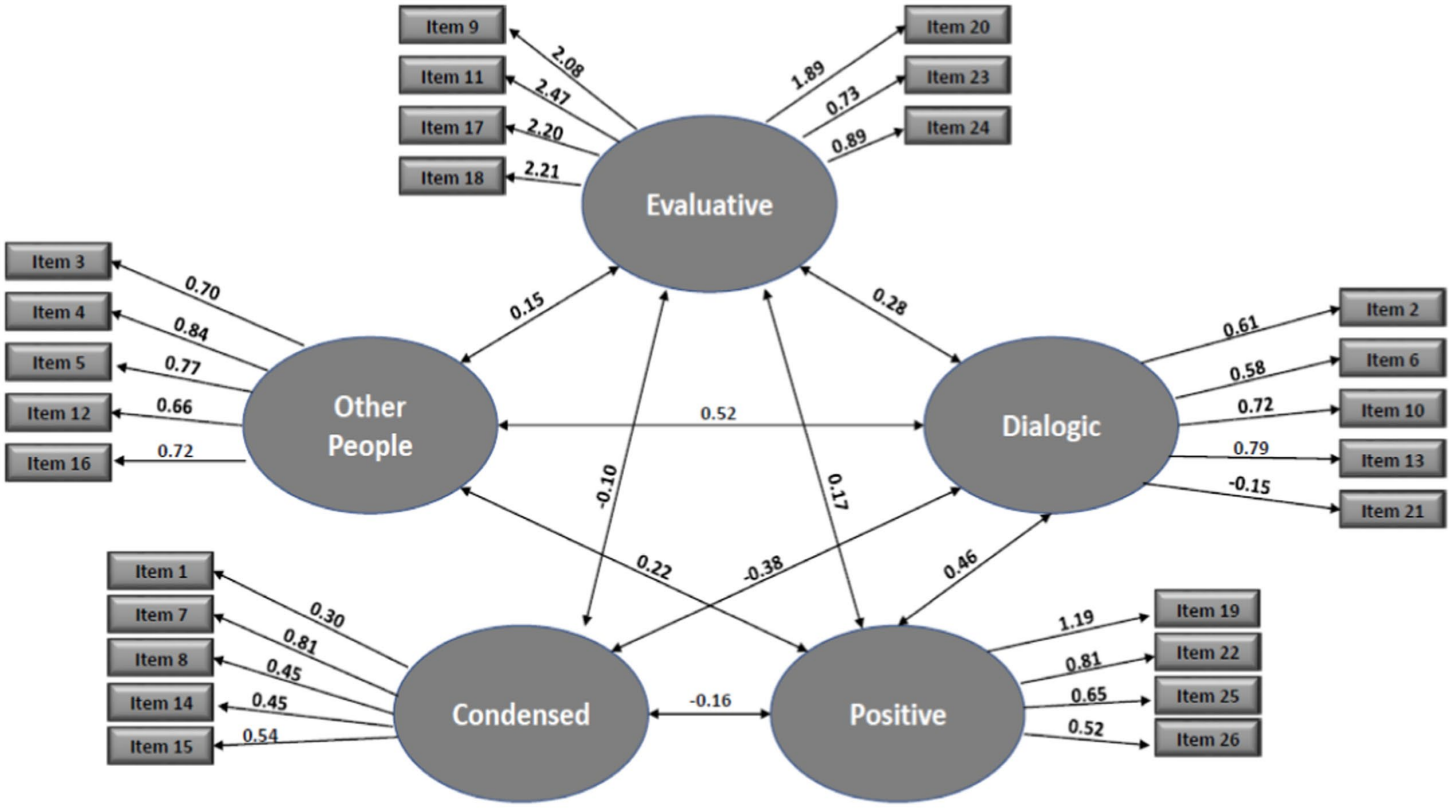
CFA of the five-factor model of the VISQ-R—Hebrew version. Ellipses indicate latent variables. Rectangles indicate observed variables. Arrows between latent variables indicate significant correlations between latent variables. Correlations between latent and observed variables were significant at *p* < 0.01.

This analysis is based on all participants (*n* = 406) and was conducted using AMOS 23.0. We chose the following values for acceptance: Comparative Fit Index (CFI) > 0.90 (Bentler and Bonett, 1980), and root mean square error of approximation (RMSEA) < 0.08 (Browne and Cudeck, 1993). The model (see Figure 1) showed good fit (Chi-square [253] = 672.57; *p* < 0.001; CFI = 90, RMSEA = 0.06). All the items loaded sufficiently onto the appropriate factors, except for item number 21 that loaded in the opposite direction than expected.

### 3.2. Reliabilities and inter-correlations of the sub-scales

The internal reliability of the subscales in the Hebrew version was acceptable, though somewhat lower than the reliabilities reported for the English version: Dialogic inner speech had an internal reliability of Cronbach’s α = 0.71; Evaluative/Critical inner speech had Cronbach’s α = 0.82; Condensed inner speech had Cronbach’s α = 0.68; Other-People in inner speech had Cronbach’s α = 0.86, and Positive/Regulatory inner speech had Cronbach’s α = 0.73. Inter-correlations of VISQ-R subscales are presented in Table 1. As seen in the table, all correlations were significant at *p* < 0.01 (two-tailed).

**Table 1:** Inter-correlations of the VISQ-R subscale-scores (*n* = 406).

	M	SD	1	2	3	4	5
1. Dialogic	4.43	1.11	-				
2. Evaluative	4.32	10.8	0.61^*^	-			
3. Other-People	2.38	1.23	0.42^*^	0.48^*^	-		
4. Condensed	3.73	0.69	0.41^*^	0.33^*^	0.28^*^	-	
5. Positive	4.64	1.10	0.47^*^	0.34^**^	0.22^*^	0.26^*^	-

* *p* < 0.01.

### 3.3. Convergent and divergent validity

Pearson’s correlations were calculated between VISQ-R subscales scores and the Hebrew version of the Behavior Rating Inventory of Executive Functions-Adult (BRIEF-A). The results based on 280 participants are shown in Table 2. All the BRIEF-A subscales and general score significantly correlated with three of the VISQ-R subscales—Dialogic, Other-People, and Evaluative/Critical, and ranging between 0.13 and 0.36, as seen in Table 2. By contrast, the Condensed and Positive/Regulatory did not yield significant correlation with any of the executive function subscales, except for significant correlation between Positive subscale of VISQ-R and Self-Monitoring subscale of BRIEF-A.

**Table 2:** Correlations of the VISQ-R subscale-scores with BRIEF-A scores (*n* = 280).

	M (SD)	Dialogic	Evaluative	Other-people	Condensed	Positive
1. Total BRIEF-A	126.81 (26.59)	0.23^**^	0.36^**^	0.27^**^	0.03	0.02
2. Inhibit	12.9 (3.37)	0.24^**^	0.30^**^	0.28^**^	0.09	0.07
3. Shifting	10.31(2.55)	0.16^**^	0.32^**^	0.25^**^	0.05	−0.04
4. Emotional control	18.47 (4.73)	0.18^**^	0.36^**^	0.24^**^	0.04	−0.05
5. Self-monitoring	8.85(2.41)	0.18^**^	0.22^**^	0.24^**^	0.00	0.12^*^
6. Working memory	13.61(3.96)	0.16^**^	0.24^**^	0.22^**^	0.00	0.01
7. Plan	16.05(4.43)	0.16^**^	0.26^**^	0.17^**^	−0.01	0.00
8. Task monitor	10.56(2.70)	0.16^**^	0.25^**^	0.20^**^	0.06	0.04
9. Organization of materials	13.14 (4.33)	0.18^**^	0.24^**^	0.20^**^	−0.02	0.09
10. Initiate	13.86(3.66)	0.15^**^	0.29^**^	0.13^**^	0.00	0.02

* *p* < 0.05;

** *p* < 0.01.

## 4. Discussion

The main objective of this study was to examine the psychometric properties of the Hebrew version of the VISQ-R (Alderson-Day et al., 2018). CFA confirmed the factor structure revealing the 5 subscales reported in the original English version (Alderson-Day et al., 2018). The confirmation of the 5 factors is evidence for structural validity of the Hebrew version of the VISQ-R. The findings further show some inter-correlations between the subscales defined by the factor analysis. In general, the inter-correlations in the current study are slightly higher than those found in the validation of the original English version, but the general pattern of the inter-correlations is quite similar (Alderson-Day et al., 2018). Dialogic inner speech was found to be most closely related to all the other factors, followed by Evaluative/Critical inner speech. The Condensed and Positive subscales show lower inter-correlation with one another and with the Other-people subscale. The internal consistency of the 5 scales supports their reliability.

The second objective of the current study was to examine the convergent and divergent validity of the Hebrew version of VISQ-R, by analyzing its relationship with the self-report questionnaire of executive functions BRIEF-A (Roth et al., 2005). The correlations between the VISQ-R and the BRIEF-A questionnaire showed clear covariance of executive functions with some subscales of the VISQ-R, and distinct variance with others. In particular, the Dialogic, Evaluative, and Other-People subscales had significant and positive correlation with all BRIEF-A subscales. By contrast, the Condensed and Positive subscales had no correlation with BRIEF-A subscales, excluding one significant correlation between Positive subscale and Self-Monitoring. These findings are partially consistent with our hypothesis. As hypothesized, the Dialogic, Other-People, and Evaluative subscales were positively correlated with difficulties in executive functions, and the Condensed subscale does not. However, the findings are inconsistent with our expectation that the Positive subscale will also be correlated with difficulties in executive functions. Although our hypothesis about the convergent and divergent validity was not fully supported, the pattern of correlations provides important evidence that the Hebrew version of the VISQ-R has good convergent and divergent validity with executive functions. In general, then, the VISQ-R in Hebrew has good psychometric properties and can be used for Hebrew speakers.

Beyond contributing to the validity of the VISQ-R in Hebrew, the pattern of correlations with the BRIEF-A is of interest as it sheds light on the functions of inner speech. Three VISQ-R subscales—Dialogic, Evaluative, and Other-people—correlated significantly with more difficulties on all aspects of executive function. This finding suggests that among individuals with difficulties in executive functions, the use of some aspects of inner speech is enhanced, possibly as a strategic compensation mechanism. This interpretation is consistent with previous findings, indicating that participants who used motivational and evaluative inner speech had reduced conflict effects and switch-effects compared to those who used inner speech less, even when controlling for intelligence and working memory abilities (Gade and Paelecke, 2019). Similar effects were observed in the context of planning tasks, where performance was negatively affected by articulatory suppression in a healthy comparison group, but not in ASD participants (Williams et al., 2012). This finding and others (for review see Petrolini et al., 2020) support the notion that although inner speech is not vital for executive function, some aspects of inner speech have an important contribution to executive functions, in particular for people with difficulties in these functions. The correlational nature of this study cannot establish primacy in time between these two processes and would be better tested in a longitudinal or an experimental design.

Another perspective on the correlations of these subscales of inner speech and executive functions is proposed by Atencio and Montero (2009). These authors build on Vygotsky’s (1987) view that inner speech is based on social collaboration with others, which, in turn, allows the child to gain the appropriate capacity to verbally mediate his own thoughts and actions. Evaluative, Dialogic, and Other-People types of inner speech involve others, directly or indirectly, whereas the other types of inner speech may happen without any representation of others. According to this view, inner speech that developed at a young age in a process of internalizing overt speech (Vygotsky, 1987) may serve individuals who experience difficulties with executive functions to regulate these difficulties through the internalization of speech that involves other people as a source of relief and reinforcement.

Two of the inner-speech subscales did not correlate with the executive function’s subscales—the Condensed and the Positive. The Condensed subscale is associated with the form of inner speech rather than its function. It reflects the use of syntactic, acoustic, and phonological language qualities besides semantics (Grandchamp et al., 2019). As such, it is not surprising that it is not correlated with executive functions. This finding is consistent with previous findings that did not observe correlations of this subscale with other psychological variables (e.g., self-esteem and dissociation, Alderson-Day et al., 2018; Depersonalization, Perona-Garcelán et al., 2017), suggesting that this subscale may be associated with processes which are inherent to the production of inner speech rather than to its psychological function in regulation and control.

The finding that the Positive subscale did not correlate with executive function (with the exception of its correlation with the self-monitoring subscale of BRIEF-A) is inconsistent with our hypothesis. This finding may be explained by the differences between the Positive subscale and the other VISQ-R subscales associated with functions of evaluation and self-regulating. For example, Shi et al. (2017) found that self-reinforcing self-talk which is similar to the Positive subscale of the VISQ-R did not have any effect on participants’ anxiety before giving a speech. This finding suggested that such self-reinforcing self-talk did not help to down-regulate the participants’ negative emotion. The authors claimed that this kind of motivational self-talk did not reduce the anxiety of participants once the task had been given, but rather it may be more relevant to situations in which self-motivation is in need of enhancement (Shi et al., 2017). As for the unique correlation of the Positive VISQ-R subscale and the Self-monitoring BRIEF-A subscale, it is plausible that the self-monitoring subscale refers to the degree to which an individual perceives him/herself as aware of the effect that his behavior has on others (Roth et al., 2005), therefore in situations that require only self-assessment and not executing an actual task, individuals who experience difficulties with their self-monitoring will tend to encourage themselves more frequently.

### 4.1. Conclusion

In conclusion, the results of the current study indicate that the factor structure of the Hebrew version of the VISQ-R (Alderson-Day et al., 2018) is consistent with the original English VISQ-R. In addition, it has good convergent and divergent validity. Hence, it can be used in future research.

The results of this study should be considered in light of its limitations. This was a cross-sectional correlational study, with a single measurement. The participants were a convenience sample, rather than a random population sample. Importantly, we used self-report rating questionnaires for convergent and divergent validity. The limitation of such questionnaires is that the participants’ responses are constrained and less ecologically valid. Future research that will use open-format questionnaires (e.g., Morin et al., 2018) may be more informative about the content of inner speech.

The aim of the current study was to adapt the VISQ-R questionnaire to Hebrew. Future research might translate the VISQ-R to other languages and cultures, to allow cross-cultural examination of its applicability, structural validity, and predictive validity. It would also be informative to further examine the distinction between the Evaluative, Dialogic, and Other People subscales and the Condensed and Positive subscales of the VISQ-R; The first three subscales are most often found to be correlated with various pathologies (for example, see Alderson-Day et al., 2018; Rosen et al., 2018; Fernyhough et al., 2019) and were correlated with EF difficulties in the current study. Are these results owed to the fact that the first three subscales involve other people or is there an alternate explanation? Finally, as always, validation of a questionnaire is best served by study in which the results at baseline on the self-report questionnaire are then tested against an experimental outcome or a longitudinal trajectory.

The use of the VISQ-R in clinical settings might be premature; however, the results of previous research and of the current study show the elevation of aspects of internal speech in various pathologies. This result should be known to clinicians. It might be useful to ask about internal speech as part of the evaluation of individuals who seek treatment for disorders such as psychosis, dissociation, or ADHD.

## Data availability statement

The raw data supporting the conclusions of this article will be made available by the authors, without undue reservation.

## Ethics statement

The studies involving human participants were reviewed and approved by the Faculty of Social and Community Sciences Institutional review board (IRB) of the Ruppin Academic Center. The patients/participants provided their written informed consent to participate in this study.

## Author contributions

DR contributed to the data curation. LL-A contributed to the formal analysis, methodology, and implementation. AZ, HK, and TS contributed to data analyses and interpretation. TS drafted the first version of the manuscript. All authors contributed to the article and approved the submitted version.

## Funding

This project was funded by the Israeli Science Foundation (grant number 216/21 to HK) and Ruppin Academic Center Research Authority Grant (grant number 33802 to HK and 33502 to AZ).

## Conflict of interest

The authors declare that the research was conducted in the absence of any commercial or financial relationships that could be construed as a potential conflict of interest.

## Publisher’s note

All claims expressed in this article are solely those of the authors and do not necessarily represent those of their affiliated organizations, or those of the publisher, the editors and the reviewers. Any product that may be evaluated in this article, or claim that may be made by its manufacturer, is not guaranteed or endorsed by the publisher.
