# Supplementary material for: The psychometric properties of the Varieties of Inner Speech Questionnaire-Revised in Hebrew
Source: Front Psychol. 2023 Jan 17;13:1092223. doi: 10.3389/fpsyg.2022.1092223 (PMC9887030; doi:10.3389/fpsyg.2022.1092223)
Supplement: Supplementary file 1 [file Data_Sheet_1.pdf]

Inner speech questionnaire (VISQ-R) in Hebrew

שם המבחן	The varieties of inner speech questionnaire – Revised (VISQ-R)
מקור	Alderson-Day, B., Mitrenga, K., Wilkinson, S., McCarthy-Jones, S., & Fernyhough, C. (2018). The varieties of inner speech questionnaire–revised (VISQ-R): replicating and refining links between inner speech and psychopathology. *Consciousness and cognition*, *65*, 48-58.
אזכור בעברית	Sabag, T., Zohar, AH., Kreiner, H., Lev-Ari, L. & Rabinowitz, D. (2023). The psychometric properties of the Varieties of Inner Speech Questionnaire-Revised in Hebrew. *Front. Psychol.* 13: 1092223.<br>doi: 10.3389/fpsyg.2022.1092223
מטרה	אפיון של דיבור פנימי וקישור בין דיבור פנימי למאפיינים פסיכופתולוגיים
סולם (מסי' פריטים+ סולם)	ישנם 5 סולמות: דיאלוג (פריטים 2, 6, 10, 13, 21), ביקורתי (פריטים 9, 11, 17, 18, 20, 23, 24), אנשים אחרים (פריטים 3, 4, 5, 12, 16), תמציתי (פריטים 1, 7, 8, 14, 15) וחיובי (פריטים 19, 22, 25, 26)
פריטים שצריך להפוך	7, 15, 21
ציינון	ממוצע של כל אחד מתתי הסולמות
תוקף הגרסה העברית	תרגום-תרגום חוזר ע"י פרופסור עדה זוהר ופרופסור חמוטל קריינר

לפנייך רשימה של היגדים. אנא סמנ.י לגבי כל אחד מהם עד כמה הוא תדיר לגבייך (1-7)

1 = כל הזמן, 7 = כל הזמן

1. אני חושב.ת לעצמי במילים ומשתמש.ת בביטויים קצרים ומילים בודדות ולא במשפטים שלמים.
2. כשאני מדבר.ת עם עצמי על דברים במחשבותיי, זה כאילו אני מתקדמ.ת הלוך וחזור, שואל.ת את עצמי שאלות ואז עונה עליהן.
3. אני שומע.ת את קולו של אדם אחר בראשי. לדוגמה, כשאני מתנהג.ת בדרך מסוימת אני שומע.ת במחשבותיי את קולה של אמי
4. אני שומע.ת את קולם של אנשים אחרים ששואלים אותי שאלות בתוך הראש שלי

5. אני שומע.ת קולות של אנשים אחרים מנדנדים לי בתוך הראש שלי
6. החשיבה שלי במילים דומה יותר לדיאלוג עם עצמי, ולא מחשבות שלי עצמי כמונולוג
7. אני חושב.ת לעצמי במילים ומשתמש.ת במשפטים שלמים
8. החשיבה שלי לעצמי במילים היא כמו הערות קצרות, ולא עברית מלאה ותקינה מבחינה דקדוקית
9. אני חושב.ת בדיבור הפנימי על מה שעשיתי והאם זה היה הדבר הנכון לעשות או לא
10. כשאני מדבר.ת עם עצמי על דברים במחשבותיי, זה כאילו אני מנהל.ת שיחה עם עצמי
11. אני מדבר.ת אל עצמי בשקט בראש ואומר.ת לעצמי לעשות דברים
12. אני שומע.ת בראשי את קולם הממשי של אנשים אחרים, אומרים דברים שהם מעולם לא אמרו לי בעבר
13. אני מדבר.ת עם עצמי הלוך ושוב במחשבותיי על דברים
14. החשיבה שלי במילים מקוצרת בהשוואה לדיבור הרגיל שלי בקול רם. לדוגמה, במקום לומר לעצמי דברים כמו 'אני צריכ.ה ללכת לקניות', אני רק אגיד לעצמי 'קניות' בראשי
15. לו הייתי רושמ.ת את מחשבותיי על הנייר, הן היו כתובות כמו משפט רגיל ותקין מבחינה דקדוקית
16. אני שומע.ת את קולם הממשי של אנשים אחרים בראשי, אומרים דברים שהם באמת אמרו לי בעבר
17. אני מדבר.ת בשקט בדיבור הפנימי שלי ואומר.ת לעצמי לא לעשות דברים מסויימים
18. אני מעריכ.ה את התנהגותי באמצעות הדיבור הפנימי שלי. לדוגמה, אני אומר.ת לעצמי, 'זה היה טוב' או 'זה היה טיפשי'
19. אני מדבר.ת עם עצמי בלב ומעודד.ת את עצמי
20. בראשי אני מדבר.ת עם עצמי באופן ביקורתי
21. מילים או משפטים מסוימים חוזרים בראשי
22. אני חושב.ת לעצמי בגוף השני, ואומר.ת דברים כמו "את.ה יכול.ה לעשות את זה" או "שכחת לעשות את זה"
23. כשאני חושב.ת במילים, זה מרגיש כאילו אני יותר מדבר.ת מאשר מקשיב.ה
24. כשאני חושב.ת במילים, זה כמו להקשיב להקלטה של הקול שלי
25. החשיבה שלי במילים היא כמו נאום או מונולוג, ולא שיחה
26. אני שולט.ת בדיבור הפנימי שלי
27. אני מרגיע.ה את עצמי בכך שאני מדבר.ת לעצמי בלב

28. מה שאני אומר.ת בדיבור הפנימי שלי גורם לי להרגיש חרד.ה
29. אני משתמש.ת במטאפורות וביטויים בדיבור הפנימי שלי, כמו "זה כזה סיוט"
30. שטף החשיבה הפנימית המילולית שלי יכול להוביל לכך שארגיש נרגש.ת מאוד
31. הדיבור הפנימי שלי תורם לכך שארגיש שאני ב"דאון" ומדוכא.ת
32. כאשר אני כועס.ת, הדיבור הפנימי שלי יכול לעזור לי להרגיע את עצמי
33. אני מופתע.ת מתוכן הדיבור הפנימי שלי
34. יש מילים או ביטויים מסוימים שאני לא מצליח.ה להוציא מראשי
35. כשאני חושב.ת לעצמי במילים על דברים מרגיזים, אני יכול.ה בקלות לשנות נושאים במחשבה שלי ולדבר עם עצמי על דברים אחרים
